# eEF1A Interacts with the NS5A Protein and Inhibits the Growth of Classical Swine Fever Virus

**DOI:** 10.3390/v7082833

**Published:** 2015-08-10

**Authors:** Su Li, Shuo Feng, Jing-Han Wang, Wen-Rui He, Hua-Yang Qin, Hong Dong, Lian-Feng Li, Shao-Xiong Yu, Yongfeng Li, Hua-Ji Qiu

**Affiliations:** State Key Laboratory of Veterinary Biotechnology, Harbin Veterinary Research Institute, Chinese Academy of Agricultural Sciences, Harbin 150001, Heilongjiang, China; E-Mails: lisu@hvri.ac.cn (S.L.); fengshuoqa@163.com (S.F.); lanyunfei901208@sina.cn (J.-H.W.); wrhe0111@163.com (W.-R.H.); ziqi317@163.com (H.-Y.Q.); dong.hong55555@163.com (H.D.); lilianfeng124603@163.com (L.-F.L.); yushaoxiong1987@hotmail.com (S.-X.Y.); yfli@hvri.ac.cn (Y.L.)

**Keywords:** classical swine fever virus, virus–host interactions, NS5A, eukaryotic elongation factor 1A, internal ribosome entry site

## Abstract

The NS5A protein of classical swine fever virus (CSFV) is involved in the RNA synthesis and viral replication. However, the NS5A-interacting cellular proteins engaged in the CSFV replication are poorly defined. Using yeast two-hybrid screen, the eukaryotic elongation factor 1A (eEF1A) was identified to be an NS5A-binding partner. The NS5A–eEF1A interaction was confirmed by coimmunoprecipitation, glutathione S-transferase (GST) pulldown and laser confocal microscopy assays. The domain I of eEF1A was shown to be critical for the NS5A–eEF1A interaction. Overexpression of eEF1A suppressed the CSFV growth markedly, and conversely, knockdown of eEF1A enhanced the CSFV replication significantly. Furthermore, eEF1A, as well as NS5A, was found to reduce the translation efficiency of the internal ribosome entry site (IRES) of CSFV in a dose-dependent manner, as demonstrated by luciferase reporter assay. Streptavidin pulldown assay revealed that eEF1A could bind to the CSFV IRES. Collectively, our results suggest that eEF1A interacts with NS5A and negatively regulates the growth of CSFV.

## 1. Introduction

Classical swine fever virus (CSFV), together with bovine viral diarrhea virus (BVDV) and border disease virus (BDV), is a member of the genus *Pestivirus* within the family *Flaviviridae* and an important pathogen of pigs that often causes huge economic losses to the pig industry worldwide. CSFV is a small enveloped virus with a single-stranded, positive-sense RNA genome of 12.3 kb. The RNA genome contains a single large open reading frame (ORF) coding for a polyprotein, flanked by a 5ʹ-untranslated region (UTR) and a 3ʹ-UTR. Under cellular and viral protease processing, the polyprotein is processed into 11 mature proteins, including four structural proteins (C, E^rns^, E1 and E2) and seven nonstructural proteins (N^pro^, p7, NS2-3, NS4A, NS4B, NS5A and NS5B) [1,2,3]. The 5ʹ-UTR contains an internal ribosome entry site (IRES) that is responsible for translation initiation of the viral genome [4]. The 3ʹ-UTR is in charge of regulating the pestiviral genome replication [5,6].

The CSFV NS5A comprises 497 amino acids and is a component of the replication complex. The conserved sequence C2717–C2740–C2742–C2767 in the NS5A protein has been proved to be important for viral growth and RNA synthesis [7]. It has been shown that NS5A regulates viral RNA replication through binding to NS5B (viral RNA-dependent RNA polymerase [RdRp]) and 3ʹ-UTR [8]. The NS5A protein of hepatitis C virus (HCV), another member of the family *Flaviviridae*, is an essential component of the replication complex [9] and plays a role in regulating cellular and viral mRNA translation [10,11]. The HCV NS5A–Core interaction is critical for the subcellular localization of NS5A and the production of infectious virus [12]. The HCV NS5A also plays an essential role in the switch from genome replication to particle assembly [13,14]. In addition, the CSFV NS5A decreases IRES-mediated translation in a dose-dependent manner and the key sites (K399, T401, E406 and L413) are also found in BVDV, BDV and HCV [15]. To date, studies have been focused on the roles of the CSFV NS5A protein in viral genomic replication and translation [8,15], but knowledge of NS5A-interacting host proteins and their impacts on CSFV replication is limited.

The eukaryotic elongation factor 1A (eEF1A), one of the most abundant protein synthesis factors, constitutes 1% to 4% of the total soluble proteins in actively dividing cells [16,17]. The canonical function of eEF1A is to bind aminoacyl-tRNA (aa-tRNA) in a GTP-dependent manner and deliver it to the A site on the ribosome during protein synthesis [18]. eEF1A is also involved in non-canonical functions including turnover of misfolded proteins, nuclear export events, binding and bundling of the actin cytoskeleton, apoptosis and the viral life cycle [19,20]. eEF1A contains three well-defined domains: the domain I binds to GTP; the domain II binds to the aminoacyl end of aa-tRNA, the domains I and II bind to the eEF1B complex, and the domain III is linked to actin binding [19].

Different groups of viruses, such as tombusvirus (TBSV), human immunodeficiency type 1 (HIV-1) and West Nile virus (WNV), utilize eEF1A as cofactor for viral transcription, translation and assembly. For TBSV, eEF1A facilitates the assembly of the TBSV replicase and stimulates minus-strand synthesis [21]. The HIV-1 Nef protein enhances the resistance to stress-induced apoptosis in primary human macrophages through the nuclear–cytoplasmic transport of eEF1A and tRNAs. Moreover, eEF1 complex subunits are critical HIV-1 reverse transcription cofactors [22,23]. In addition, the BVDV NS5A was also shown to interact with eEF1A [24]. Considering CSFV, BVDV, HCV and WNV are all *Flaviviridae* members, we suppose that the CSFV NS5A protein possibly interacts with eEF1A or affects the CSFV growth in host cells. In this study, we identified the host protein eEF1A as a novel NS5A-interacting partner that negatively regulates the growth of CSFV.

## 2. Results

### 2.1. NS5A Interacts with eEF1A

**Figure 1:**
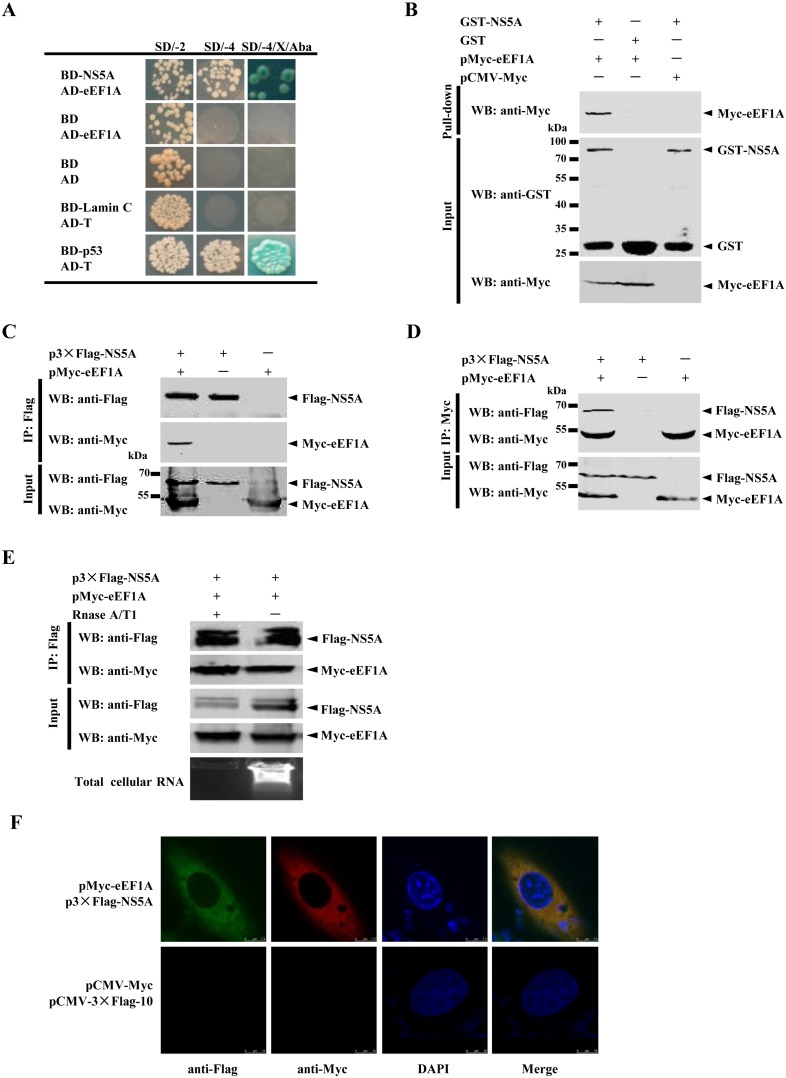
Interaction of NS5A and eEF1A. (**A**) Reactivity of NS5A with eEF1A in a yeast two-hybrid (Y2H) system. Yeast Y2HGold strain was cotransformed with pGBKT7-NS5A (BD-NS5A) as a bait and pGADT7-eEF1A (AD-eEF1A) as a prey. As a positive control, pGBKT7-p53 (BD-p53) and pGADT7-T (AD-T) were cotransformed into Y2HGold strain. Cotransformation of pGBKT7-Lamin C (BD-Lamin C) and AD-T was used as a negative control. All the yeast colonies cotransformed with the above plasmids were grown on synthetically defined (SD) medium lacking His and Leu (SD/-2), SD medium lacking His, Leu, Trp and Ade (SD/-4) and SD/-4 medium containing 5-bromo-4-chloro-3-indolyl-α-D-galactopyranoside (X-α-Gal) and aureobasidin A (Aba) (SD/-4/X-α-Gal/Aba); (**B**) Glutathione S-transferase (GST) pulldown assay. The GST or GST-NS5A fusion proteins expressed in *Escherichia coli* BL21(DE3) were purified with glutathione Sepharose 4B resin and incubated with the lysate of HEK293T cells overexpressing the Myc-tagged eEF1A. After washing with cold PBS, the bound proteins were subjected to sulfate-polyacrylamide gel electrophoresis (SDS-PAGE) (10%) and Western blotting using the anti-GST polyclonal antibody (PAb) (1:2000) and the anti-Myc monoclonal antibody (MAb) (1:1000); (**C**) Coimmunoprecipitation (Co-IP) analysis of Myc-tagged eEF1A and Flag-tagged NS5A. HEK293T cells were cotransfected with the indicated plasmids (+) or empty vectors (-) for 48 h. The transfected cells were lyzed and incubated with a mouse anti-Flag MAb, followed by incubation with the Protein G-Agarose for 6 h at 4 °C. The immunoprecipitate was analyzed by Western blotting using the anti-Flag MAb (1:1000) and a rabbit anti-Myc PAb (1:500); (**D**) Co-IP analysis of eEF1A and NS5A by the anti-Myc MAb. HEK293T cells were cotransfected with the indicated plasmids (+) or empty vectors (−), the cell lysate was collected at 48 h post-transfection (hpt), incubated with the anti-Myc MAb and Protein G-Agarose. The immunoprecipitate was examined by Western blotting using the anti-Flag MAb (1:1000) and the anti-Myc PAb (1:500); (**E**) Effects of RNase A/T1 treatment on the NS5A–eEF1A interaction. Extracts of HEK293T cells overexpressing different proteins were treated or untreated with RNase A/T1 for 1 h prior to Co-IP and immunoblotted with the indicated antibodies. The total RNA in the immunoprecipitate was visualized by ethidium bromide-staining; (**F**) Colocalization of NS5A protein with eEF1A. HEK293T cells were cotransfected with p3×Flag-NS5A and pMyc-eEF1A. Cells were fixed at 48 hpt and subjected to indirect immunofluorescence assay to detect 3×Flag-NS5A (green) and Myc-eEF1A (red) with mouse anti-Flag and rabbit anti-Myc antibodies. The position of the nucleus is indicated by DAPI (blue) staining in the merged image. Samples were imaged and magnified 630 times on the Leica SP2 confocal system fitted with a 63× objective lens. Scale bar = 7.5 μm.

In the yeast two-hybrid screen, only one protein, eEF1A, was identified to be a potential binding protein for NS5A (Figure 1A). To confirm the interaction between NS5A and eEF1A, glutathione S-transferase (GST) pulldown assay was performed with the GST-tagged NS5A protein expressed in *Escherichia coli* and the Myc-tagged eEF1A protein expressed in HEK293T cells. The results showed that GST-NS5A but not GST interacts with eEF1A (Figure 1B). To further verify the interaction between NS5A and eEF1A, coimmunoprecipitation (Co-IP) assay was performed with HEK293T cells overexpressing 3×Flag-tagged NS5A and Myc-tagged eEF1A. After incubation with anti-Flag monoclonal antibody (MAb) and Protein G-Agarose, the Myc-tagged eEF1A was found to coprecipitate with the 3×Flag-tagged NS5A (Figure 1C). Furthermore, the 3×Flag-tagged NS5A was shown to coprecipitate with Myc-eEF1A when the cell lysate was incubated with anti-Myc MAb and Protein G-Agarose (Figure 1D). Considering that both eEF1A and NS5A show a strong affinity for nucleic acids, we investigated whether the NS5A–eEF1A interaction might be mediated through a nonspecific RNA bridge. The results showed that the NS5A–eEF1A interaction was not influenced by RNase treatment, indicating that the interaction is not due to nonspecific RNA-mediated binding (Figure 1E). To examine the colocalization of NS5A protein with eEF1A, the subcellular localization of 3×Flag-NS5A and Myc-eEF1A was examined by confocal microscopy. Both 3×Flag-NS5A and Myc-eEF1A were colocalized in the cytoplasm (Figure 1F). Taken together, the results demonstrated that eEF1A is an interacting partner of the CSFV NS5A protein.

### 2.2. The Domain I of eEF1A is Critical for the NS5A–eEF1A Interaction

**Figure 2:**
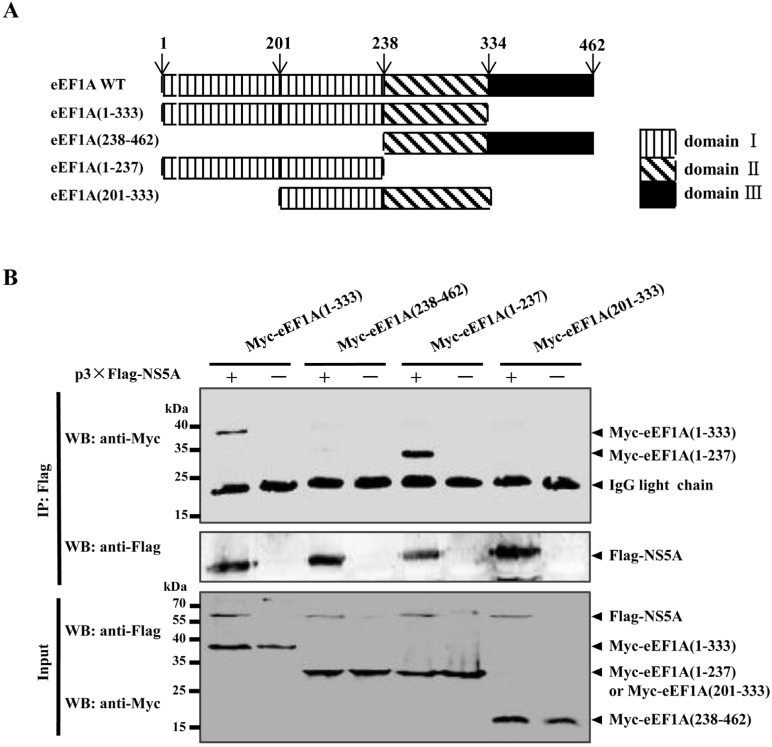
The domain I of eEF1A is required for its association with NS5A. (**A**) Schematic representation of eEF1A protein domains and the individual eEF1A deletion mutants tested in this study; (**B**) Coimmunoprecipitation (Co-IP) analysis of the association of 3×Flag-NS5A with Myc-eEF1A mutants in cotransfected HEK293T cells.

To determine which domain(s) of eEF1A is responsible for the association with NS5A, a series of Myc-tagged deletion mutants of eEF1A were constructed (Figure 2A) and tested for the interaction with NS5A by Co-IP assay. The results showed that eEF1A(1-133) and eEF1A(1-237) were capable of binding NS5A (Figure 2B), indicating that the domain I is critical for the NS5A–eEF1A interaction.

### 2.3. Overexpression of eEF1A Inhibits CSFV Replication

**Figure 3:**
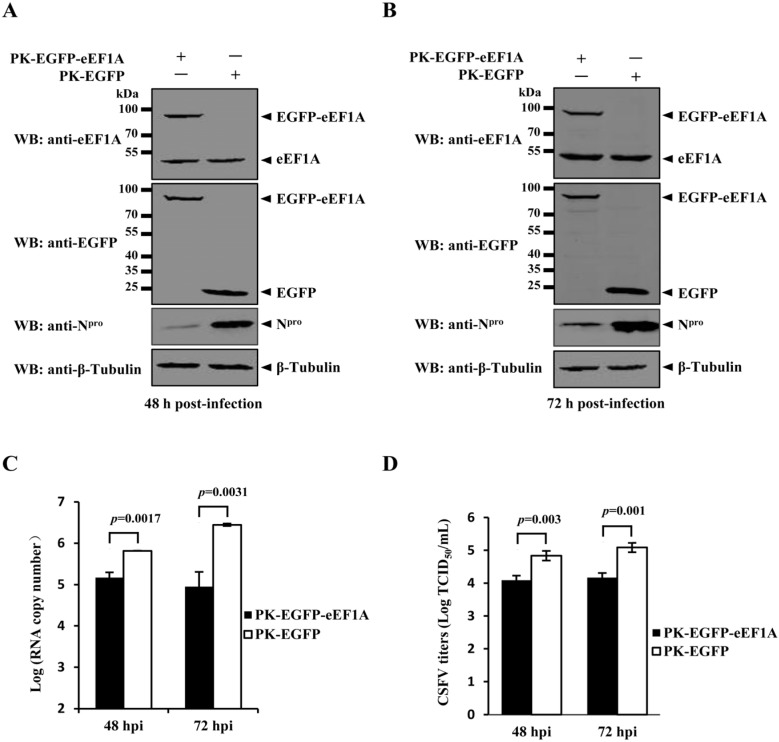
Overexpression of eEF1A suppresses the CSFV growth. (**A** and **B**) Western blotting analysis of overexpressed EGFP-eEF1A and CSFV N^pro^. PK-15 cells were transduced with the lentivirus expressing EGFP-eEF1A or EGFP, followed by infection with CSFV at a multiplicity of infection (MOI) of 0.1. Protein expression was examined by Western blotting at 48 (**A**) and 72 h (**B**) post-infection (hpi) using a rabbit anti-eEF1A monoclonal antibody (MAb) (1:2000) and a mouse anti-N^pro^ polyclonal antibody (PAb) (1:500) (produced in-house). β-Tubulin served as an internal control; (**C**) Real-time RT-PCR analysis of CSFV genomic copy numbers. The culture supernatant of the CSFV-infected cells at 48 and 72 hpi were harvested and evaluated for the viral genomic copy numbers by real-time RT-PCR as described previously [25]. (**D**) CSFV titers in eEF1A-overexpressed cells. Virus titers in the supernatant were determined at 48 and 72 hpi and expressed as 50% tissue culture infective doses (TCID_50_)/mL. Error bars represent the standard deviations of the means from three independent experiments. *p-*values were indicated above the bars.

To investigate the effects of eEF1A on the CSFV replication, stable PK-15 cell line overexpressing EGFP-eEF1A (PK-EGFP-eEF1A) or EGFP (PK-EGFP) was generated and infected with the CSFV Shimen strain at a multiplicity of infection (MOI) of 0.1. The N^pro^ expression in the CSFV-infected PK-EGFP-eEF1A cells was decreased at 48 and 72 h post-infection (hpi), compared with that in the PK-EGFP cells (Figure 3A,B). Real-time RT-PCR analysis revealed that the viral genome copies in the PK-EGFP-eEF1A cells were also reduced at 48 and 72 hpi, compared with the PK-EGFP cells (Figure 3C). Additionally, yields of infectious virus in culture supernatant of PK-EGFP-eEF1A cells were decreased at 48 and 72 hpi (Figure 3D). The results indicated the eEF1A negatively modulates the CSFV replication.

### 2.4. Knockdown of eEF1A by Lentivirus-Mediated shRNAs Enhances CSFV Replication

**Figure 4:**
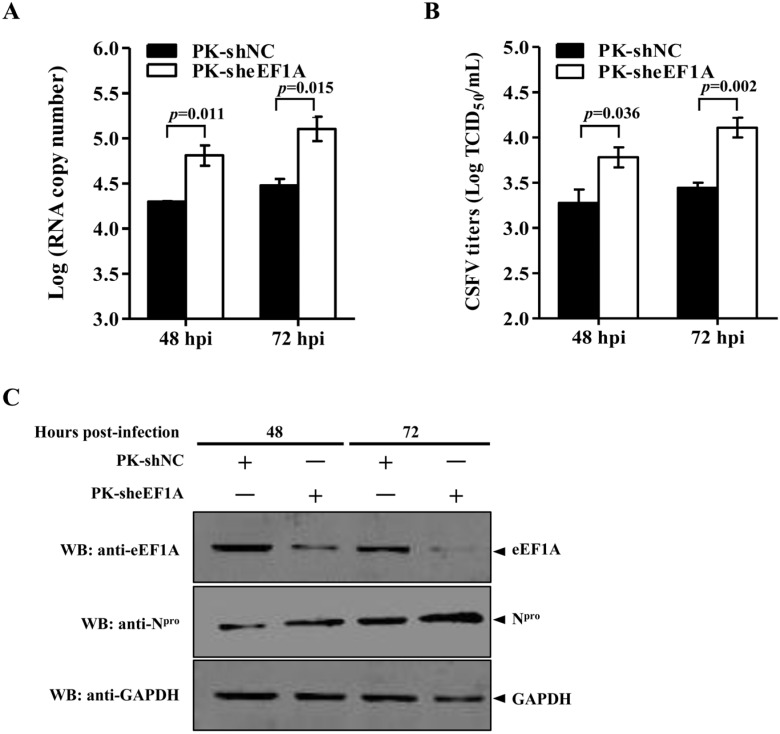
Knockdown of eEF1A increases CSFV growth. (**A**) CSFV genome copies in eEF1A knockdown cells. PK-15 cells transduced with the lenti-sheEF1A or lenti-NC for 24 h were infected with the CSFV Shimen strain at a multiplicity of infection (MOI) of 0.1 for 48 h or 72 h. The CSFV genome copy numbers were assessed using a real-time RT-PCR assay as described previously [25]. (**B**) CSFV titers in eEF1A-knocked down cells. Virus titers in the supernatant were determined at 48 and 72 h post-infection (hpi) and expressed as 50% tissue culture infective doses (TCID_50_)/mL. Error bars represent the standard deviations of the means from three independent experiments. *p-*values were indicated above the bars. (**C**) Knockdown of eEF1A by lentivirus-mediated shRNAs increased expression of the CSFV N^pro^ protein. The endogenous eEF1A and the CSFV N^pro^ protein were detected by Western blotting using an anti-eEF1A monoclonal antibody (MAb) and an anti-N^pro^ polyclonal antibody (PAb), respectively.

To further determine the effects of eEF1A on CSFV replication, recombinant lentiviruses expressing short hairpin RNA (shRNA) against eEF1A (sheEF1A) or non-targeting negative control shRNA (shNC) were generated and transduced into PK-15 cells, resulting in efficient knockdown of the eEF1A expression (Figure 4A). Twenty-four hours after lentivirus-mediated shRNAs transduction, PK-15 cells were infected with the CSFV Shimen strain at an MOI of 0.1. The viral replication was analyzed by real-time RT-PCR, virus titration and Western blotting. In comparison with the shNC control, the viral genome copies in the eEF1A-knocked down cells were increased at 48 and 72 hpi (Figure 4A). Similarly, the CSFV titers were significantly increased after eEF1A knockdown in PK-15 cells (Figure 4B), and the N^pro^ protein expression in the sheEF1A-transduced cells was increased at 48 and 72 hpi (Figure 4C). The results indicate that the eEF1A negatively modulates the CSFV replication.

### 2.5. eEF1A Reduces the Translation Efficiency of CSFV IRES 

**Figure 5:**
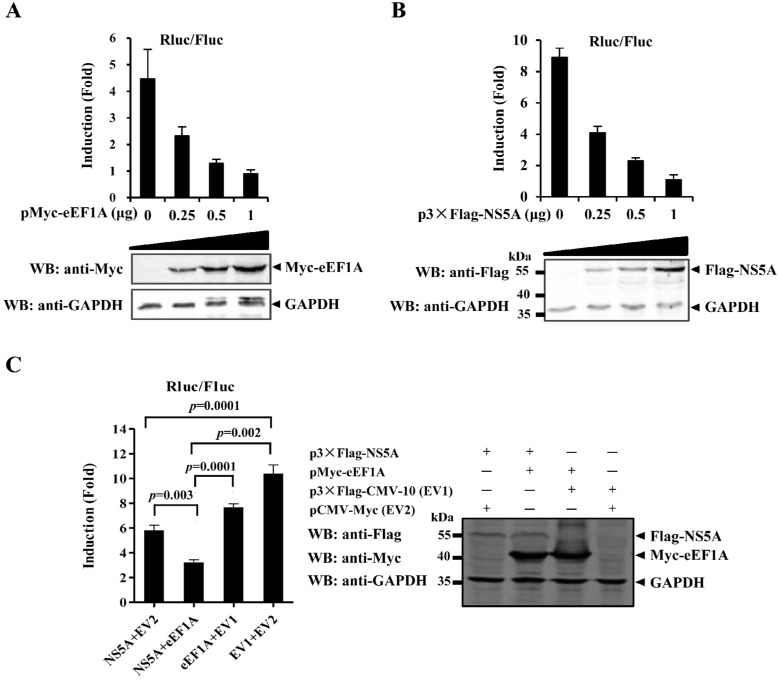
Overexpressed eEF1A or/and NS5A inhibits the CSFV internal ribosome entry site (IRES) activity. (**A**) eEF1A inhibitory effect on the CSFV IRES activity in a dose-dependent manner. The plasmids pMyc-eEF1A (0, 0.25, 0.5 or 1 μg), pFluc/IRES/Rluc (0.75 μg) and pLXSN-T7 (0.3 μg) were cotransfected into HEK293T cells. The reporter gene activity was detected and expressed as fold induction. Protein expression of Myc-tagged eEF1A was examined by Western blotting using a rabbit anti-Myc polyclonal antibody (PAb) (1:500); (**B**) NS5A inhibitory effect on the CSFV IRES activity in a dose-dependent manner. The plasmids p3×Flag-NS5A (0, 0.25, 0.5 or 1 μg), pFluc/IRES/Rluc (0.75 μg) and pLXSN-T7 (0.3 μg) were cotransfected into HEK293T cells. The reporter gene activity was detected and presented as fold induction. Western blotting was performed using a mouse anti-Flag monoclonal antibody (MAb) (1:1000) to verify the expression of 3×Flag-NS5A; (**C**) Effects of eEF1A on the NS5A-mediated inhibition of the CSFV IRES activity. Examination of the CSFV IRES activity using a luciferase reporter assay. Luciferase reporter plasmids pFluc/IRES/Rluc (0.75 μg) and pLXSN-T7 (0.3 μg) were cotransfected into HEK293T cells with or without p3×Flag-NS5A (0.5 μg) and pMyc-eEF1A (0.5 μg). The reporter gene activity was detected and presented as fold induction. To prove the expression of 3×Flag-NS5A and Myc-eEF1A, Western blotting was performed using anti-Flag (1:1000) and anti-Myc (1:500) antibodies. GAPDH was included as an internal control. Rluc level represented the CSFV IRES activity. The Fluc gene under the control of the T7 promoter was used as an internal control. *p-*values were indicated above the bars. The data were averaged from six replicates of two independent experiments.

A previous study showed that NS5A decreases the CSFV IRES-mediated translation in a dose-dependent manner [13]. To examine the effects of eEF1A on the NS5A-mediated inhibition of the CSFV IRES activity, the luciferase reporter assay was used in this study. The results showed that eEF1A (Figure 5A), as well as NS5A (Figure 5B), inhibited the CSFV IRES activity in a dose-dependent manner. To further investigate the inhibitory effects of eEF1A and NS5A coexpression, eEF1A- and NS5A-expressing plasmids were cotransfected with the luciferase reporter plasmids into HEK293T cells. The results indicated that the eEF1A did not antagonize the inhibition of the IRES activity by NS5A when NS5A and eEF1A were coexpressed (Figure 5C).

**Figure 6:**
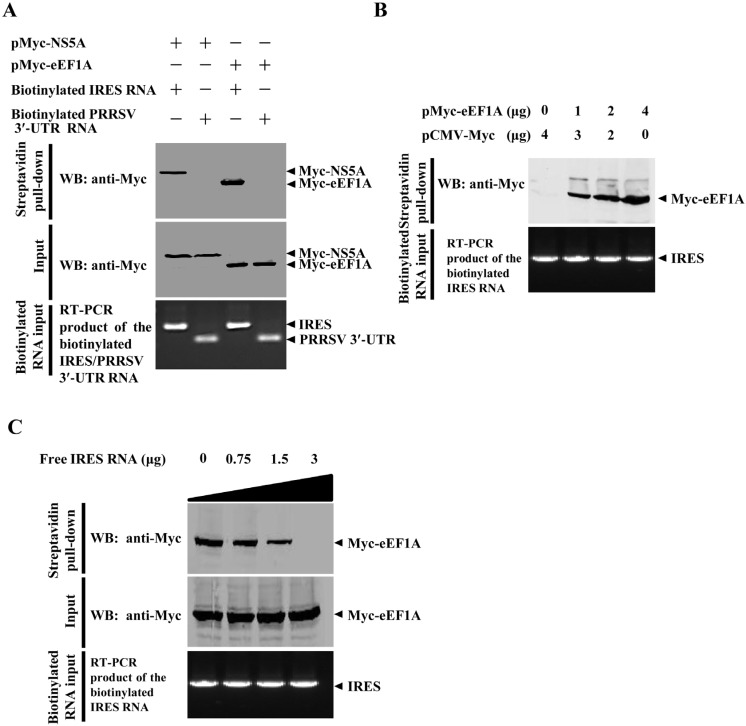
eEF1A binds to the CSFV internal ribosome entry site (IRES). (**A**) eEF1A as well as NS5A interacts with the CSFV IRES. HEK293T cells were transfected with pMyc-eEF1A or pMyc-NS5A. The cell lysate was incubated with the biotinylated CSFV IRES or porcine reproductive and respiratory syndrome virus (PRRSV) 3ʹ-UTR, followed by incubation with streptavidin beads. The bound proteins were analyzed by immunoblotting using the anti-Myc monoclonal antibody (MAb) (1:1000); (**B**) eEF1A interacts with the CSFV IRES in a dose-dependent manner. HEK293T cells grown in the 6-well plate were transfected with an increased amount of pMyc-eEF1A (0, 1, 2 and 4 μg), followed by streptavidin pulldown assay as described above; (**C**) Competitive RNA pulldown assay. HEK293T cells grown in the 6-well plate were transfected with the indicated pMyc-eEF1A (4 μg). The cell lysate was incubated with an increased amount of the IRES RNA (0, 0.75, 1.5 and 3.0 μg) for 1 h at 4 °C and incubated with the biotinylated IRES RNA and streptavidin beads. The bound proteins were subjected to immunoblotting using the anti-Myc MAb.

### 2.6. eEF1A Interacts with the CSFV IRES

The observation that overexpression of eEF1A resulted in the inhibition of the CSFV IRES activity prompted an examination of the interaction between eEF1A and IRES of CSFV. Streptavidin pulldown assay showed that the biotinylated CSFV IRES, but not the biotinylated porcine reproductive and respiratory syndrome virus (PRRSV) 3ʹ-UTR, could bind to eEF1A in a dose-dependent manner (Figure 6A,B). Competitive RNA pulldown assay indicated that the association of eEF1A with the CSFV IRES could be inhibited by the unlabeled CSFV IRES (Figure 6C). The results demonstrated that eEF1A binds to the CSFV IRES and inhibits its activity.

## 3. Discussion

The CSFV NS5A functions as the modulation of viral RNA replication via interacting with NS5B and 3ʹ-UTR [8]. In addition, the NS5A protein can inhibit the IRES-mediated translation through binding to the IRES located in the 5ʹ-UTR. A previous study showed that the NS5A-interacting cellular proteins were screened using Y2H analysis, and the identified proteins are mostly related to gene transcription, protein degradation, protein folding and metabolism [26]. Recently, heat shock protein 70 has been identified to interact with the CSFV NS5A and enhance viral RNA replication [27]. In this study, eEF1A was identified as a novel NS5A partner and a negative regulator of CSFV replication. eEF1A had not been identified in the previous study, probably due to the different cell types (endothelial cells *vs.* primary macrophages) used to construct the cDNA library [26]. We showed that NS5A and eEF1A were colocalized in the cytoplasm. As NS5A is the component of the replication complex, whether eEF1A is also localized in the replication complex needs further investigation.

The domain I of eEF1A was shown to be critical for the NS5A–eEF1A interaction, implying that it may be a potential target of anti-CSFV strategies. We also showed that eEF1A was able to bind to the IRES located in the 5ʹ-UTR and inhibit its activity. In addition, we investigated the effects of eEF1A on the inhibition of the IRES activity by NS5A; however, we demonstrated that eEF1A did not antagonize the inhibition of the IRES activity by NS5A, indicating that NS5A and eEF1A perhaps bind to distinct parts of the IRES.

Several viral proteins have been observed to associate with eEF1A. The nucleocapsid protein of severe acute respiratory syndrome coronavirus (SARS-CoV) could modulate cell cytokinesis and proliferation via interacting with eEF1A [28]. eEF1A also interacts with the nucleocapsid protein of transmissible gastroenteritis virus (TGEV) and regulates the virus replication [29]. Furthermore, eEF1A can be utilized as the cofactors of the viral RNA synthesis for several viruses, such as HIV-1 [22,23], TBSV [21] and WNV [30]. Previous studies on turnip mosaic virus (TuMV), tobacco mosaic virus (TMV) and TBSV have shown that eEF1A interacts primarily with the viral RdRp complex, and thus acts as a chaperone and supports viral replication [31,32,33]. As members of *Flaviviridae*, both BVDV NS5A and HCV NS4A interact with eEF1A [24,34]. Thereinto, the exact role of eEF1A on the BVDV replication remains unclear [24]. The HCV NS4A inhibits the viral IRES-mediated translation, and the translation inhibitory effect of NS4A was relieved by the addition of purified recombinant eEF1A in the *in vitro* translation system [34]. However, we showed here that eEF1A negatively regulates the CSFV growth. We conclude that the different roles of eEF1A in viral replication may vary depending on the distinct interactions between eEF1A and different viruses. In this study, the eEF1A was shown to bind to the IRES and inhibit its activity, thus it is possible to suppress the CSFV replication.

Since eEF1A binds to aa-tRNA among mammalian RNA species [35], it is possible for eEF1A to bind to the similar RNA structures located in 5ʹ- or 3ʹ-UTR of the viral genome. It has been shown that interaction between eEF1A and the 3ʹ-terminal stem-loop of the WNV genomic RNA facilitates the viral minus-strand RNA synthesis [30,36,37]. In the present study, we demonstrated that eEF1A binds to IRES located in the 5ʹ-UTR of CSFV. Whether eEF1A binds to 3ʹ-UTR of CSFV and influences RNA replication requires further study. We showed that eEF1A inhibits CSFV growth and reduces IRES activity of CSFV. Thus, we speculate that eEF1A affects the process of the viral protein translation via interacting with the CSFV IRES.

In summary, we demonstrated that eEF1A interacts with NS5A and negatively modulates the CSFV growth. The possible mechanism of the negative effect of eEF1A on the CSFV growth is that eEF1A interacts with the CSFV IRES and inhibits its activity. Further studies are needed to define the detailed interplay among eEF1A, NS5A and IRES in the life cycle of CSFV.

## 4. Materials and Methods

### 4.1. Cells, Viruses and Virus Titration Assay

PK-15 or SK6 cells (porcine kidney cell lines) and HEK293T cells were grown in Dulbecco’s modified Eagle’s medium (DMEM) supplemented with 10% fetal bovine serum (FBS) confirmed to be free of BVDV and anti-BVDV antibodies. The CSFV Shimen strain was propagated in PK-15 or SK6 cells. Viral titers in the culture supernatant of CSFV-infected cells were determined as described previously [38].

**Table 1:** Primers used in this study.

Primers	Sequences (5ʹ→3ʹ)	Usage
3×FLAG-NS5A-FP	GCGAATTCATCAAGTAATTACATTCTAGAGC	Amplification of NS5A
3×FLAG-NS5A-RP	ACGGATCCTTACAGTTTCATAGAATACAC	
Myc-NS5A-FP	GAATTCATTCAAGTAATTACATTC	Amplification of NS5A
Myc-NS5A-RP	ATCTCGAGTTACAGTTTCATAGAATACACTTTTGCA	
GST-NS5A-FP	CGGAATTCATGTCAAGTAATTACATTCTAGAGCTCC	Amplification of NS5A
GST-NS5A-RP	ATCTCGAGTTACAGTTTCATAGAATACACTTTTGCA	
Myc-eEF1A-FP	CGAGATCTATATGGGAAAGGAGAAGACTCACATC	Amplificationof eEF1A
Myc-eEF1A-RP	ATCTCGAGTTATCATTTAGCCTTCTGAGC	
Myc-eEF1A-FP(1-333)	CGAGATCTATATGGGAAAGGAGAAGACTCACATC	Amplification of eEF1A mutant(1-333)
Myc-eEF1A-RP(1-333)	ATCTCGAGTTATGGGTCATTTTTGCTGTCACC	
Myc-eEF1A-FP(1-237)	CGAGATCTATATGGGAAAGGAGAAGACTCACATC	Amplification of eEF1A mutant(1-237)
Myc-eEF1A-RP(1-237)	ATCTCGAGTTATGGTAGAATGCAATCCAGAGC	
Myc-eEF1A-FP(238-462)	CGAGATCTATATGCCAACTCGTCCAACTGACAAGC	Amplification of eEF1A mutant(238-462)
Myc-eEF1A-RP(238-462)	CGAGATCTATATGGGAAAGGAGAAGACTCACATC	
Myc-eEF1A-FP(201-333)	CGAGATCTATATGCTGGAGCCAAGTGCTAATATG	Amplification of eEF1A mutant(201-333)
Myc-eEF1A-RP(201-333)	ATCTCGAGTTATGGGTCATTTTTGCTGTCACC	
FUGW-eEF1A-FP	ACAGGCCATTACGGCCATGGGAAAGGAGAAGACTC	Amplification of eEF1A
FUGW-eEF1A-RP	TACGGCCGAGGCGGCCTTATCATTTAGCCTTCTGAGC	

### 4.2. Construction of Expression Vectors

The swine eEF1A gene (GenBank accession no. NM_001097418.2) and its various domains were amplified by PCR and cloned into the pMyc vector (Clontech, Palo Alto, CA, USA) to generate pMyc-eEF1A, pMyc-eEF1A(1-333), pMyc-eEF1A(1-237), pMyc-eEF1A(238-462) and pCMV-Myc-eEF1A(201-333), respectively. The CSFV NS5A gene (GenBank accession no. EU497410.1) was amplified by PCR and cloned into the p3×Flag-CMV-10 vector (Sigma-Aldrich, ST. Louis, MO, USA) or pCMV-Myc to generate p3×Flag-NS5A or pMyc-NS5A. The primers for amplifying eEF1A and NS5A genes are listed in Table 1.

### 4.3. Yeast Two-Hybrid Screen

A Matchmaker two-hybrid system (catalog no. 630489; Clontech) was used to screen host proteins that interact with NS5A. Briefly, the bait construct pGBKT7-NS5A (BD-NS5A) was transformed into the yeast strain Y2HGold and hybridized with a cDNA library derived from porcine macrophages [39]. Transformants were selected for growth on synthetically defined (SD) medium lacking His, Leu, Trp and Ade (SD/-4) for 3 to 6 days at 30 °C. The clones were then transferred to SD/-4 medium containing 5-bromo-4-chloro-3-indolyl-α-D-galactopyranoside (X-α-Gal) and aureobasidin A (Aba) (SD/-4/X-α-Gal/Aba). Blue colonies were selected and inoculated with 5 mL of SD/-4 medium for shaking culture at 30 °C for 1 to 3 days. Yeast plasmids were extracted using a yeast plasmid kit (catalog no. D3376; Omega, Guangzhou, China) according to the manufacturer’s instructions, and the target inserts were verified by sequencing using the Gal4 AD and 3ʹAD primers. To validate the interaction between NS5A and the cellular proteins, the bait and prey plasmids were cotransformed into the Y2HGold yeast strain using a yeast transformation system 2 (catalog no. 630439; Clontech). The interaction between murine p53 and SV40 large T-antigen was used as a positive control, and the human lamin C protein, which does not interact with the SV40 large T-antigen, was included as a negative control.

### 4.4. Plasmid Transfection

HEK293T cells grown in 6-well plates (Nunc, Waltham, MA, USA) were cotransfected with 5 µg of pMyc-eEF1A and 3 µg of p3×Flag-NS5A using an X-tremeGENE HP DNA transfection reagent (catalog no. 06366236001; Roche, Penzberg, Germany) according to the manufacturer’s instructions. Briefly, the cells were transfected with 400 µL of serum-free DMEM containing 8 µg of plasmids and 12 µL of X-tremeGENE HP DNA transfection reagent. At 6 h post-transfection (hpt), the transfection mixture was replaced with DMEM supplemented with 10% FBS and incubated for an additional 48 h before being assayed.

### 4.5. GST Pulldown Assay

For expression of the GST-tagged NS5A protein, the NS5A gene was subcloned into the pGEX-6p-1 vector (catalog no. 28-9546-48; GE Healthcare, Piscataway, NJ, USA), creating pGEX-NS5A. The GST or GST-NS5A fusion protein expressed in *E. coli* BL21(DE3) were purified with the glutathione-Sepharose 4B resin (catalog no. 10049253; GE Biosciences, Piscataway, NJ, USA) according to the manufacturer’s instructions. Briefly, the expression of GST or GST-NS5A protein was induced by addition of 1 mM isopropylthiogalactoside (IPTG) (catalog no. ST098; Beyotime, Shanghai, China).The bacterial cells were harvested and resuspended in cold phosphate-buffered saline (PBS) containing 1 mg/mL protease inhibitor cocktail (catalog no. 11873580001; Roche, Penzberg, Germany), followed by mild sonication. Insoluble components were removed by centrifugation at 10,000 ×*g* for 20 min. Subsequently, the soluble GST or GST-NS5A protein was incubated with the glutathione-Sepharose 4B resin for 4 h at 4 °C. The resins were washed four times with cold PBS and incubated with 300 μL of the lysate of the HEK293T cells transfected with pMyc-eEF1A for 2 h at 4 °C. After an extensive wash with PBS, the bound proteins were separated by sodium dodecyl sulfate-polyacrylamide gel electrophoresis (SDS-PAGE) followed by immunoblotting using the anti-Myc MAb (1:1000) (catalog no. M4439; Sigma-Aldrich) and an anti-GST polyclonal antibody (PAb) (1:2000) (catalog no. AB101; Tiangen, Beijing, China).

### 4.6. Coimmunoprecipitation Assay

For Co-IP assay, the p3×Flag-NS5A and pMyc-eEF1A were cotransfected into HEK293T cells as described above. The cells were collected at 48 hpt, washed two times with cold PBS, and lyzed with the CHAPS lysis buffer (1% CHAPS, 150 mM NaCl, 5 mM MgCl_2_ and 10 mM Tris-HCl [pH 7.5]) containing 1 mM PMSF (catalog no. ST506; Beyotime) and 1 mg/mL protease inhibitor cocktail at 4 °C for 1 h. The cell lysate was centrifuged at 13,000 ×*g* for 20 min at 4 °C. The clarified lysate was treated with a mixture of RNase A (25 U/mL) (catalog no. 2158; TaKaRa, Dalian, China) and RNase T1 (1 U/µL) (catalog no. PF0059; Fermentas, Burlington, Canada) for 1 h at room temperature prior to Co-IP analysis. The lysate was precleared with the Protein G-Agarose (catalog no. 11243233001; Roche) at 4 °C for 4 h followed by incubation with the indicated antibodies at 4 °C for 4 h, and the Protein G-Agarose was then added and incubated at 4 °C for 2 h. The Protein G-Agarose was washed three times with PBS, and the bound proteins were separated by SDS-PAGE followed by Western blotting using the indicated antibodies.

### 4.7. Confocal Microscopy

HEK293T cells were cotransfected with pMyc-eEF1A (3 μg) and p3×Flag-NS5A (2 μg). After 48-h incubation, the cells were fixed with 4% paraformaldehyde in PBS for 30 min and permeabilized with 0.1% Triton X-100 for 15 min. The cells were then incubated with an anti-Myc PAb (1:100) (catalog no. C3956; Sigma-Aldrich) or anti-Flag MAb (1:100) (catalog no. F1804; Sigma-Aldrich) for 2 h. The cells were incubated with goat anti-mouse IgG (whole molecule)-fluorescein isothiocyanate (FITC) antibody (1:100) (catalog no. F2012; Sigma-Aldrich) and goat anti-rabbit IgG (whole molecule)-tetramethyl rhodamine isocyanate (TRITC) antibody (1:100) (catalog no. T6778; Sigma-Aldrich). Subsequently, the cells were stained with 4,6-diamidino-2-phenylindole (DAPI) (1:1000) (catalog no. D8417; Sigma-Aldrich) for 15 min and examined using a Leica SP2 confocal system (Leica Microsystems, GmbH, Wetzlar, Germany).

### 4.8. Construction of a Stable Cell Line Overexpressing eEF1A

To construct a stable cell line overexpressing eEF1A, the swine eEF1A gene was subcloned into the lentivirus vector pFUGW (Addgene, Cambridge, MA, USA). HEK293T cells were cotransfected with the recombinant plasmid pFUGW-eEF1A or the empty vector pFUGW, and the packaging plasmids psPAX2 (Addgene) and pMD2.G (Addgene). At 6 hpt, the medium was replaced with DMEM containing 5% FBS. After 48-h incubation, the supernatant of the cell culture was harvested and ultra-centrifuged to concentrate the recombinant lentiviruses. Subsequently, PK-15 cells grown on 6-well plates were transduced with the lentiviruses of 10 transduction units (TU) per cell. The expression of EGFP-eEF1A in the transduced PK-15 cells was examined by Western blotting using a rabbit anti-eEF1A MAb (1:2000) (catalog no. ab157455; Abcam, Cambridge, UK).

### 4.9. Gene Knockdown by shRNAs

To knock down the expression of eEF1A in PK-15 cells, a lentivirus vector-mediated shRNA targeting eEF1A was constructed. Briefly, the upper and lower sequences of shRNAs for eEF1A and non-targeting negative control (NC) are listed in Table 1. The oligonucleotides were annealed and cloned into the lentivirus vector pLVX-shRNA2 (Clontech). The resulting recombinant plasmids pLVX-eEF1A-shRNA1, pLVX-eEF1A-shRNA2 or pLVX-NC-shRNA were cotransfected with the packaging plasmids psPAX2 and pMD2.G into HEK293T cells in 10-cm cell dishes. At 48 hpt, the lentiviruses in the culture supernatant were concentrated at 6000 ×*g* for 20 min at 4 °C using an Amicon Ultra-15 centrifugal filter unit with Ultracel-100 membrane (catalog no. UFC910096; Millipore, Billerica, MA, USA). The lentiviuses were titrated and transduced into PK-15 cells as described above. Knockdown of eEF1A was validated by Western blotting using the anti-eEF1A MAb (1:2000).

### 4.10. Real-Time RT-PCR

Total RNA was extracted from the culture supernatant of CSFV-infected PK-15 cells using a TRIzol reagent (catalog no. 15596026; Invitrogen, Waltham, MA, USA). The isolated RNA was then reverse transcribed to cDNA with reverse transcriptase XL (AMV) (catalog no. 2621; TaKaRa, Dalian, China) according to the manufacturer’s instructions. Genomic copies of CSFV were quantified by a real-time RT-PCR assay as described previously [25]. The experiment was carried out in triplicates.

### 4.11. Luciferase Reporter Assay

In luciferase reporter assay, the reporter plasmid pFluc/IRES/Rluc [40], a gift from Prof. Belsham, was used, which contains the firefly luciferase (Fluc) gene under the control of the T7 promoter and the renilla luciferase (Rluc) gene under the control of the CSFV IRES. The plasmid pLXSN-T7 expressing the T7 RNA polymerase [41] was also used in the assay. For the luciferase reporter assay, HEK293T cells grown on 24-well plates were cotransfected with 750 ng of pFluc/IRES/Rluc, 300 ng of pLXSN-T7 and different amounts of p3×Flag-NS5A or pMyc-eEF1A. After 48-h incubation, the reporter gene activity was analyzed with a dual-luciferase reporter assay system (catalog no. E1910; Promega, Madison, WI, USA) and measured with the TD-20/20 Luminometer (Turner Designs, Morgan Hill, CA, USA) according to the manufacturer’s instructions. The data represent the Rluc activity normalized to the Fluc activity. Three independent experiments were carried out in duplicates.

### 4.12. Streptavidin Pulldown Assay

The CSFV IRES or PRRSV 3ʹ-UTR fragment was amplified and cloned into the pcDNA3.1(+) vector to generate pcDNA-IRES or pcDNA-PRRSV-3ʹ-UTR. Subsequently, the plasmids were linearized by *Eco*RV and transcribed *in vitro* using a RiboMAX™ large scale RNA production system (catalog no. P1300; Promega) according to the manufacturer’s instructions. The resulting RNA was labeled with photobiotin (catalog no. A14216; Baoman, Shanghai, China) using a mercury vapor lamp for 30 min. HEK293T cells grown on 6-well plates were transfected with the indicated plasmids (pMyc-NS5A and pMyc-eEF1A) and lyzed with the lysis buffer. The lysate collected from two wells of the 6-well plate (lyzed with 150 μL of lysis buffer per well) was incubated with the biotinylated IRES RNA (2 μg) for 4 h at 4 °C, and then incubated with streptavidin beads (catalog no. 11205D; Invitrogen) for another 30 min at room temperature. The beads were washed three times with lysis buffer and analyzed by immunoblotting with the anti-Myc MAb (1:1000). For competitive RNA pulldown, the cell lysate was incubated with an increased amount of unlabeled IRES RNA (0, 0.75, 1.5 and 3 μg) for 1 h at 4 °C and then incubated with the biotinylated IRES and streptavidin beads. The experiments were carried out in triplicates.

### 4.13. Statistical Analysis 

Statistical analysis was performed using the SPSS 17.0 software. Student’s *t*-test or one-way analysis of variance was used to compare the CSFV genomic copies. A *p*-value of <0.05 was considered significant.
